# Identification and Biological Validation of a Chemokine/Chemokine Receptor-Based Risk Model for Predicting Immunotherapeutic Response and Prognosis in Head and Neck Squamous Cell Carcinoma

**DOI:** 10.3390/ijms24043317

**Published:** 2023-02-07

**Authors:** Ye Wang, Shimeng Wang, Houshang Wang, Jin Yang, Hongmei Zhou

**Affiliations:** State Key Laboratory of Oral Diseases, National Center of Stomatology, National Clinical Research Center for Oral Diseases, Frontier Innovation Center for Dental Medicine Plus, West China Hospital of Stomatology, Sichuan University, Chengdu 610041, China

**Keywords:** head and neck squamous cell carcinoma, chemokine and chemokine receptor, risk model, tumor microenvironment, immunotherapy, prognosis

## Abstract

Over 80% of head and neck squamous cell carcinoma (HNSCC) patients failed to respond to immunotherapy, which can likely be attributed to the tumor microenvironment (TME) remolding mediated by chemokines/chemokine receptors (C/CR). This study aimed to establish a C/CR-based risk model for better immunotherapeutic responses and prognosis. After assessing the characteristic patterns of the C/CR cluster from the TCGA-HNSCC cohort, a six-gene C/CR-based risk model was developed to stratify patients by LASSO Cox analysis. The screened genes were multidimensionally validated by RT-qPCR, scRNA-seq, and protein data. A total of 30.4% of patients in the low-risk group had better responses to anti-PD-L1 immunotherapy. A Kaplan–Meier analysis showed that patients in the low-risk group had longer overall survival. A time-dependent receiver operating characteristic curve and Cox analyses indicated that risk score served as an independent predictive indicator. The robustness of the immunotherapy response and prognosis prediction was also validated in independent external datasets. Additionally, the TME landscape revealed that the low-risk group was immune activated. Furthermore, the cell communication analysis on the scRNA-seq dataset revealed that cancer-associated fibroblasts were the main communicators within the C/CR ligand–receptor network of TME. Collectively, The C/CR-based risk model simultaneously predicted immunotherapeutic response and prognosis, potentially optimizing personalized therapeutic strategies of HNSCC.

## 1. Introduction

Head and neck squamous cell carcinoma (HNSCC) is the seventh most common cancer worldwide, with 850,000 new cases in 2020 [1,2,3]. More than 60% of HNSCC patients in the advanced stage develop relapse or metastasis, representing a poor prognosis with a mortality rate of over 50% [1]. The emergence of immunotherapies, especially immune checkpoint inhibitor (ICI) therapy, has achieved clinical benefits in HNSCC patients. Among them, anti-programmed cell death protein 1 (PD-1) agent pembrolizumab monotherapy or combination therapy has been approved as the first line for relapse/metastasis HNSCC, and it prolongs the median overall survival (OS) by between 0.8 months and 2.3 months [4,5]. Nevertheless, anti-PD-1 therapy failed to induce a response in 82% to 87% of patients [5]. It is reported that diverse factors might contribute to the low response rate of immunotherapy, including a complex mutation landscape [6,7], heterogeneity of the tumor microenvironment (TME) [8], etc.

Predicting immunotherapeutic response and prognosis before clinical therapeutics will identify target subpopulations for selective screening, and it will ultimately achieve personalized therapy to improve therapeutic outcomes. Previous research showed that the traditional TNM stage could not meet the demand of accurately designing clinical strategies and forecasting clinical outcomes in the era of immunotherapy because patients with the same TNM stage might exhibit different responses to treatment and prognosis [9,10]. With the development of high-throughput sequencing technology and bioinformatics, an increasing number of risk models have been developed, including tumor-mutation-signature-based or one single-gene-signature-based risk models [11,12]. Notably, most of them focused on the intrinsic characteristics of the tumor or stroma [13,14]. However, the dynamic and complex relationships of the TME are also worth exploring. For instance, by spatial transcriptomics sequencing and single-cell RNA sequencing (scRNA-seq) [15,16], recent research led to an improved understanding of the complexity of the TME space and the effects of cell–cell communication within the TME.

**Figure 1:**
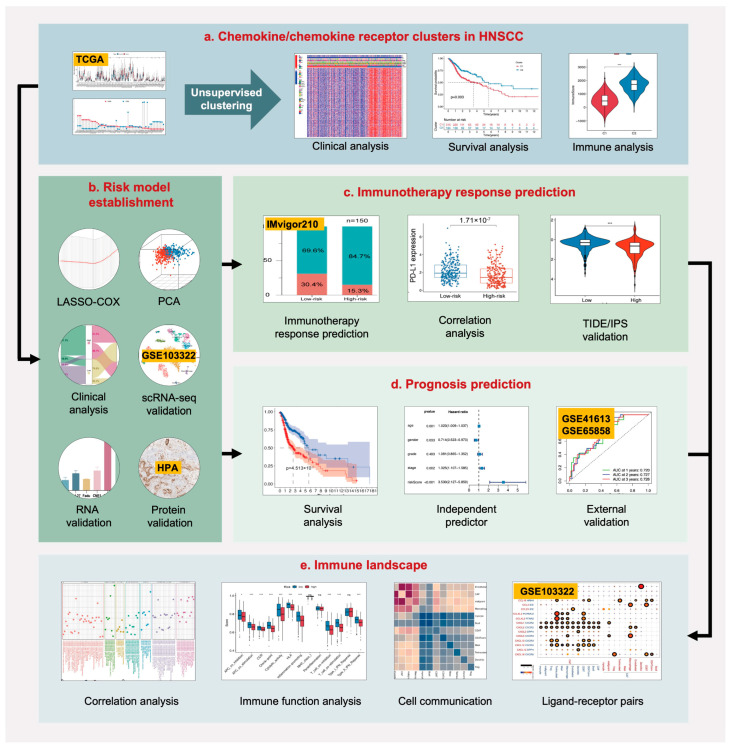
The workflow of our research.

Chemokines/chemokine receptors (C/CR) are crucial components of the TME [17]. By regulating complex and dynamic cell communication between the tumor and stromal cells, they serve as pivotal controllers and influence TME heterogeneity and the immunotherapy response [8,18]. Given that C/CR work cooperatively as a complex system, the interaction of multiple genes causes stronger biological effects than a single gene. A risk model based on the whole C/CR signature in HNSCC has not yet been reported. Therefore, this study comprehensively overviewed the genetic and immune characteristics of the chemokine superfamily in HNSCC. We further developed a multiple-gene risk model to predict the immunotherapy response and prognosis of HNSCC patients simultaneously. Experimental validation and multi-omics analysis combined with the Gene Expression Omnibus (GEO) datasets and scRNA-seq were performed to verify the reliability of the model and analyze the potential mechanism. The workflow of our research is shown in Figure 1.

## 2. Results

### 2.1. Chemokine/Chemokine Receptor Clusters in the TCGA-HNSCC Cohort

**Figure 2:**
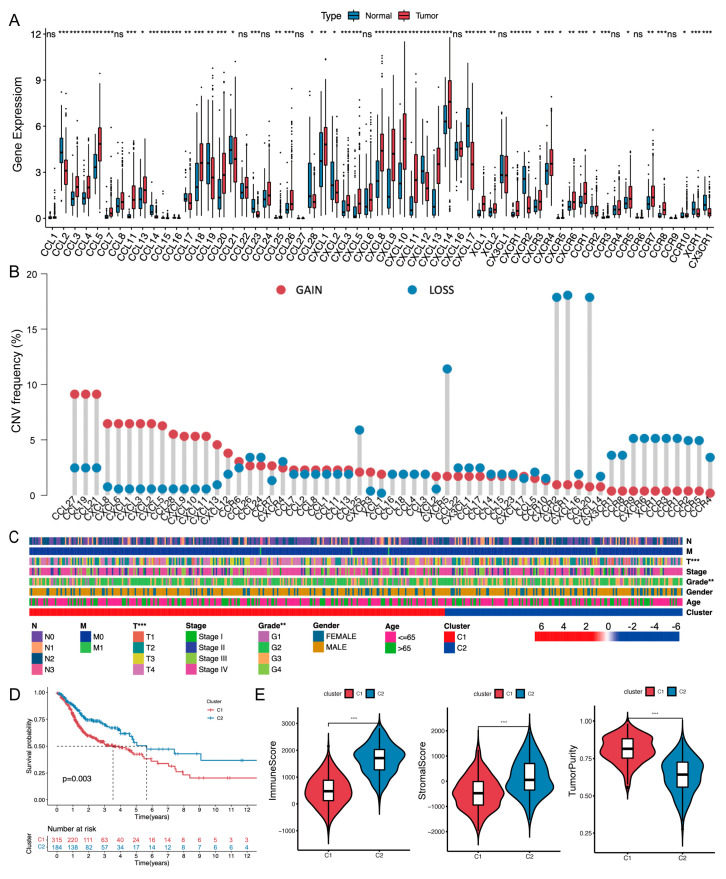
Identification of chemokine/chemokine receptor clusters in the TCGA-HNSCC cohort. (**A**) The expression level of C/CR genes between normal and HNSCC tissues. (**B**) CNV frequencies of C/CR genes in the TCGA-HNSCC cohort. (**C**) Different clinicopathological features between the two clusters. (**D**) Kaplan–Meier survival analysis of the two clusters. (**E**) Immune score, stromal score, and tumor purity of the two clusters. ns, *p* > 0.05, * *p* < 0.05, ** *p* < 0.01, *** *p* < 0.001.

The human chemokine superfamily consists of 67 C/CR genes, including 45 ligands and 22 receptors [19]. We systematically analyzed their molecular characteristics in The Cancer Genome Atlas (TCGA)-HNSCC cohort. The expression of 27 genes was significantly elevated in tumors, whereas CCL19, CCL21, CXCL12, etc. were reduced (Figure 2A). Next, we explored copy number variation (CNV) frequencies and somatic mutation. The data on CNV frequency indicated a prevalent CNV alteration of C/CR (Figure 2B). For example, CCL19 possessed an amplification alteration with a frequency of 10%, while CCL20 possessed a deletion alteration with a frequency of 18%. The location of CNV alteration on the 23 chromosomes was shown in Figure S1A. The DNA mutation data of C/CR showed that only 47 patients (9.29%) experienced mutation, with missense mutations and C>T as the main mutation styles (Figure S1B). In total, we discovered that the C/CR cluster displayed different expression levels and genetic landscapes in the TCGA-HNSCC cohort.

Subsequently, based on the expression level of the 67 C/CR genes, 436 TCGA-HNSCC patients were classified into two clusters by unsupervised clustering analysis (Figure S1C). Figure 2C and Figure S1D depict the two clusters with different clinicopathological features, in which T staging and histological grading showed a remarkable difference (*p* < 0.001 and *p* < 0.01, respectively). Survival analysis revealed that cluster two was associated with a favorable prognosis (*p* = 0.003; Figure 2D). To distinguish the TME features between cluster one and cluster two, we found that the pathways enriched in cluster two were closely linked to immune-related pathways, including antigen processing and presentation, T/B cell receptor signaling pathways, chemokine signaling pathways, etc. (Figure S1E). In addition, the TME characteristics were evaluated by the ESTIMATE algorithm and single-sample gene-set enrichment analysis (ssGSEA). Figure 2E showed that cluster two had a higher immune score and stromal score as well as a lower score in tumor purity than cluster one (*p* < 0.001). Figure S1F indicates that cluster two was characterized by higher immune cell infiltration and immune function compared with cluster one. Taken together, cluster two with an immune-activated phenotype was associated with a favorable prognosis, suggesting that the C/CR-cluster-based classification method is reasonable to help risk stratification in HNSCC patients.

### 2.2. Establishment and Validation of a Chemokine/Chemokine Receptor-Based Risk Model

**Figure 3:**
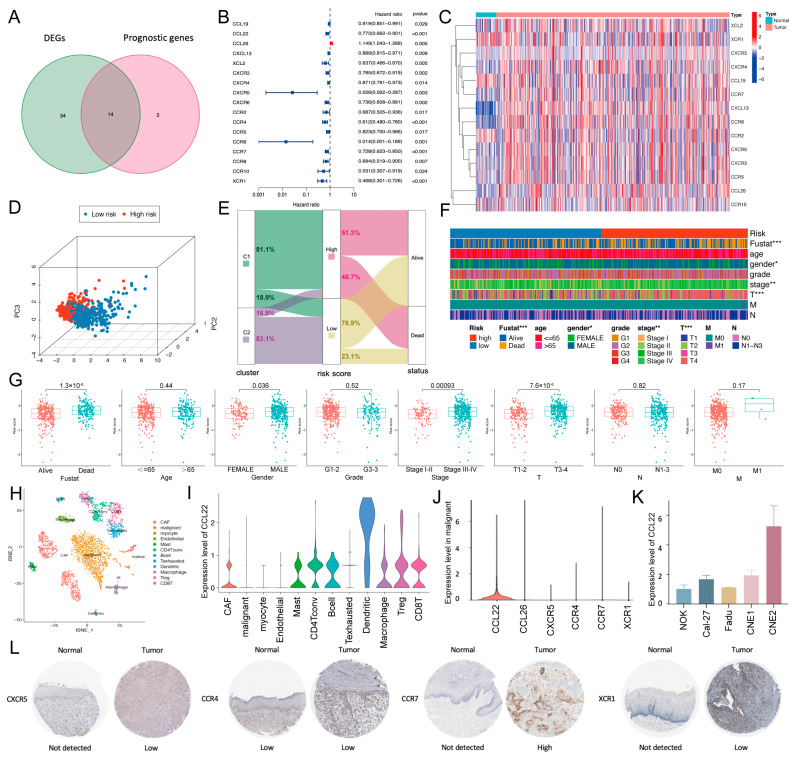
Establishment and validation of the risk model. (**A**) Venn diagram to identify DEGs related to prognosis. (**B**) Hazard ratio of the prognostic genes. (**C**) Heatmap of the overlapping genes. (**D**) PCA analysis showed a remarkable difference between the low- and high-risk groups. (**E**) Alluvial diagram of cluster distributions in groups with different risk scores and survival status. (**F**) Different clinicopathological features of the two risk groups. (**G**) The risk score in different groups was divided by clinicopathological features. (**H**) t-SNE classified cell clusters based on transcriptome data. (**I**) Expression level of CCL22 among 12 cell types in scRNA-seq. (**J**) Expression levels of the six risk genes in malignant cells in scRNA-seq. (**K**) Relative expression levels of CCL22 in five cell lines by RT-qPCR. (**L**) The protein expression levels in the HPA database (CCL22 and CCL26 not found). ns, *p* > 0.05, * *p* < 0.05, ** *p* < 0.01, *** *p* < 0.001.

To simplify the 67-gene C/CR cluster, a risk model was established. Univariate COX analysis showed that 14 differentially expressed genes (DEGs) were related to prognosis (Figure 3A). For instance, the risk ratio of CCL26 was 1.149 (95% CI = 1.043–1.266, *p* = 0.005; Figure 3B), indicating that elevated expression of CCL26 was associated with a better prognosis. The heatmap further delineated the expression level of the 14 overlapping genes (Figure 3C). Via least absolute shrinkage and selection operator (LASSO)-Cox regression, 6 of 14 genes were finally concluded, including CCL22, CCL26, CXCR5, CCR4, CCR7, and XCR1 (Figure S2A). The risk score was calculated as follows: risk score = (−0.080828341018676 * expression of CCL22) + (0.113331803768543 * expression of CCL26) + (−1.17804112684914 * expression of CXCR5) + (−0.0766717829119386 * expression of CCR4) + (−0.0320017408770535 * expression of CCR7) + (−0.311888198969236 * expression of XCR1). TCGA-HNSCC patients were stratified into a low-risk group (n = 245) and a high-risk group (n = 247). Principal component analysis (PCA) and the distribution of patients confirmed that the two subgroups could be distinguished (Figure 3D,E). Then the distinct clinicopathological features were displayed (Figure 3F,G). Male gender (*p* = 0.036), tumor stage III-IV (*p* < 0.001), and T3-4 patients (*p* < 0.001) were significantly related to a higher risk score.

To validate the cellular distribution of the six genes within the TME, a single-cell dataset with 18 HNSCC patients was selected. After quality control (Figure S2B), 3000 stromal cells were clustered into 12 major clusters by t-distributed stochastic neighbor embedding (t-SNE) dimensionality reduction clustering (Figure 3H). First, we measured the expression of the six genes in stromal cells and malignant cells and found that CCL22 possessed a prevalent higher expression in the 12 cell clusters (Figure 3I and Figure S2C). In malignant cells, the expression of CCL22 was also prominent among the six genes (Figure 3J). In the HNSCC cell line CNE2, the expression of CCL22 was also elevated compared with that in a normal oral keratinocyte cell line, as assessed by real-time quantitative polymerase chain reaction (RT-qPCR) (*p* < 0.05; Figure 3K). Protein expression of screened genes was verified in the Human Protein Atlas (HPA) database (Figure 3L). Hence, we believed that the model was reliable.

### 2.3. Therapeutic Response Prediction and Validation of the Risk Model

**Figure 4:**
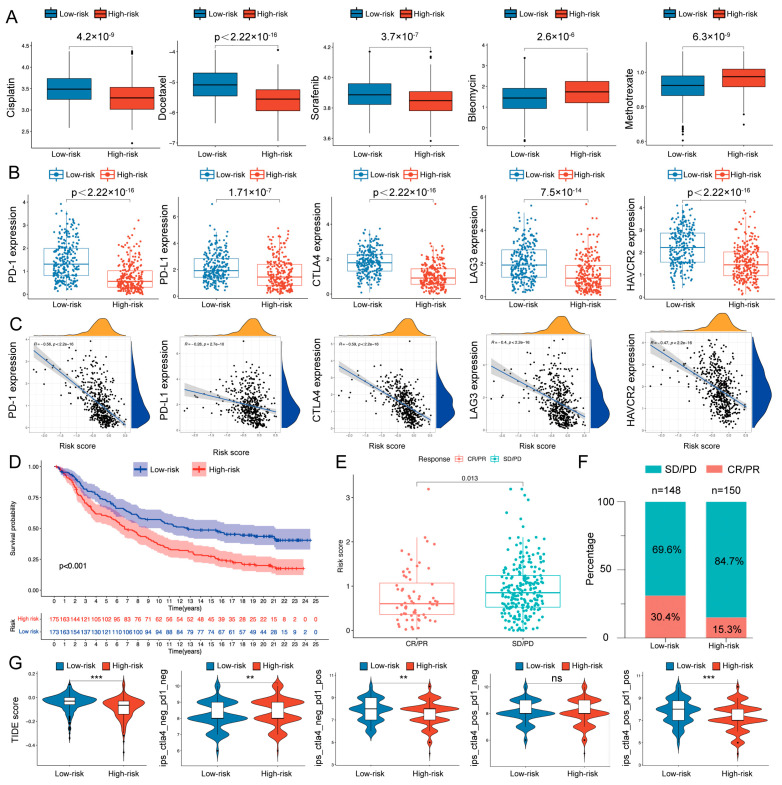
Therapeutic benefit prediction and validation. (**A**) The differences in the estimated IC50 levels of cisplatin, docetaxel, sorafenib, bleomycin, and methotrexate between the two groups. (**B**) Expression levels of PD-1, PD-L1, CTLA4, LAG3, and HAVCR2 in the two risk groups. (**C**) Correlation analysis between risk score and the ICI expression level. (**D**) Survival analysis for low- and high-risk groups in the anti-PD-L1 immunotherapy cohort. (**E**) Risk scores of patients with different clinical responses. (**F**) Percentage of response (CR/PR) and non-response (SD/PD) patients in the two risk groups. (**G**) TIDE score and IPS score reflected the response to ICIs. ns, *p* > 0.05, * *p* < 0.05, ** *p* < 0.01, *** *p* < 0.001.

We next examined whether the C/CR-based risk model could predict the clinical response of HNSCC patients. First, we compared the differences in the estimated half maximal inhibitory concentration (IC50) levels of five chemotherapy drugs commonly used (Figure 4A) to evaluate chemosensitivity. The low-risk group exhibited a higher IC50 level of cisplatin, docetaxel, and sorafenib (*p* < 0.001), while the high-risk group exhibited an increased IC50 level of bleomycin and methotrexate (*p* < 0.001). Furthermore, to evaluate the prediction performance for ICI responses, we analyzed the relationship between risk scores and the expression levels of ICIs. Compared with the high-risk group, the low-risk group showed a significantly higher expression of PD-1, PD-L1, CTLA4, LAG3, and HAVCR2 (*p* < 0.001; Figure 4B,C). Then, based on the anti-PD-L1 immunotherapy cohort IMvigor210, we found that patients with low-risk scores exhibited a significant prolonged OS (*p* < 0.001; Figure 4D) and better response to therapy (*p* = 0.013, Figure 4E; *p* = 0.0023, Figure 4F). The Tumor Immune Dysfunction and Exclusion (TIDE) and immunophenoscore (IPS) score were further constructed to quantitatively evaluate the effectiveness of ICIs. As Figure 4G illustrates, the TIDE, IPS, IPS-PD1, and IPS-PD1-CTLA4 scores were higher in the low-risk group, indicating that the low-risk group might respond better to immunotherapy, especially anti-PD-1 therapy and anti-PD-1 combined with anti-CTLA-4 therapy. Collectively, the results indicated that the risk model based on the six C/CR genes could predict responses to immunotherapies.

### 2.4. Prognosis Prediction and Validation of the Risk Model

**Figure 5:**
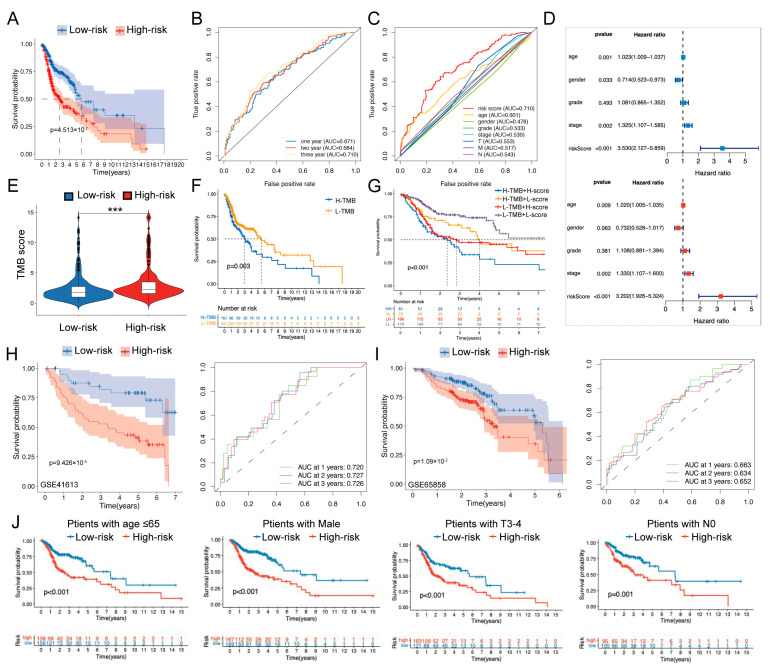
Prognosis prediction and validation. (**A**) Kaplan–Meier survival analysis for low- and high-risk groups. (**B**) ROC curves to predict the sensitivity and specificity of one-, two-, and three-year survival times based on the risk score. (**C**) ROC curves to compare the prognostic accuracy of risk score, age, gender, grade, tumor stage, and TNM in three-year survival time. (**D**) Univariate (up) and multivariate (down) Cox analyses to evaluate whether the risk score was an independent prognostic factor. (**E**) TMB score of two risk groups. (**F**) Kaplan–Meier survival analysis of the high- and low-TMB score groups. (**G**) Kaplan–Meier survival analysis for patients stratified by both TMB and risk scores. (**H**) Validation of survival analysis in GSE41613. (**I**) Validation of survival analysis in GSE65858. (**J**) Analysis of OS based on risk scores stratified by age, gender, and TNM stages in the TCGA-HNSCC cohort. ns, *p* > 0.05, * *p* < 0.05, ** *p* < 0.01, *** *p* < 0.001.

A total evaluation of clinicopathological features and a Kaplan–Meier analysis showed that the prognosis of patients in the low-risk group was significantly better than that in the high-risk group (*p* < 0.001; Figure 3G and Figure 5A). Area under the curve (AUC) values of receiver operating characteristic (ROC) curves reached 0.671 at one year, 0.684 at two years, and 0. 710 at three years (Figure 5B). Meanwhile, the three-year ROC curve showed a better capacity for predicting prognosis when compared with other clinicopathological features, including the TNM stage (Figure 5C). Univariate and multivariate Cox analyses were also employed to determine if that risk score was an independent prognostic factor (Figure 5D). Recent studies have demonstrated that high tumor mutation burden (TMB) was associated with poor prognosis in HNSCC [7]. Considering the prognostic effects of TMB, we further examined the potential relationship between TMB and our risk model. We found that TMB values were significantly higher in the high-risk group (*p* < 0.001; Figure 5E), and a Kaplan–Meier analysis showed a consistent result indicating that the high-TMB group was associated with poorer prognosis (*p* = 0.003; Figure 5F). Thus, we evaluated the synergistic effect of TMB and risk scores in the prognostic stratification of HNSCC. Either in the high- or low-TMB subgroups, the high-risk group was associated with a worse prognosis (Figure 5G). As shown in the mutation waterfall plots, the TP53 gene showed the highest mutation rate (53% vs. 71% in the low- and high-risk groups, respectively; Figure S3A). Collectively, the risk score served as a reliable and independent predictive indicator.

To verify the prognostic performance of the risk model, we performed external validation. First, both low-risk groups of the two datasets (GSE41613 and GSE65858) exhibited significantly longer OS than the high-risk groups (Figure 5H,I). The AUC analysis at one, two, and three years showed that the risk score still had good prognosis prediction efficiencies. In addition, stratified survival analysis was performed to explore whether the risk score retained its predictive ability in different clinical subgroups. A Kaplan–Meier analysis showed that OS was significantly prolonged in the low-risk group in the subgroups of age ≤ 65, male, T3-4, and N0 (*p* ≤ 0.001; Figure 5J and Figure S3B). Collectively, the results confirmed the accuracy and reliability of the C/CR-based risk model for prognosis prediction, and the low-risk group possessed a favorable prognosis.

### 2.5. TME and Single-Cell Landscape of the Risk Model

**Figure 6:**
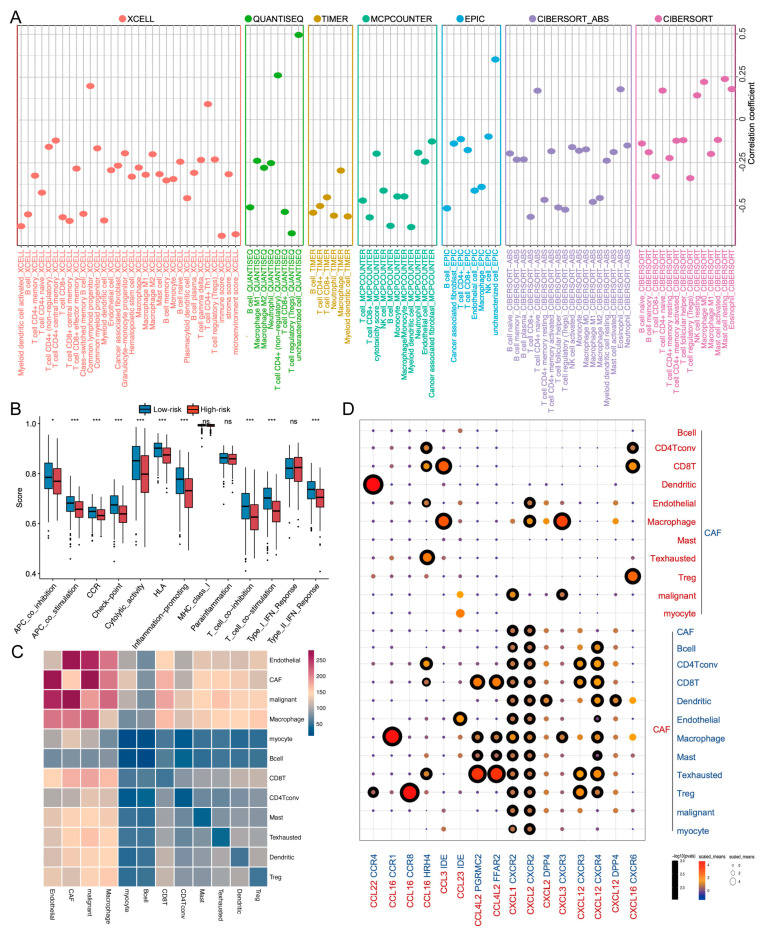
TME and single-cell landscape. (**A**) Correlation between risk score and immune cell infiltration via XCELL, QUANTISEQ, TIMER, MCPCOUNTER, EPIC, CIBERSORT_ABS, and CIBERSORT. (**B**) Immune function analysis of the two risk groups by ssGSEA. (**C**) The number of potential ligand–receptor pairs between cell groups predicted by CellphoneDB. (**D**) Ligand–receptor pairs of chemokines and receptors between CAFs and other cell groups (solid sphere with black around it is defined as a statistical difference; solid sphere without black around it indicates no statistical difference). ns, *p* > 0.05, * *p* < 0.05, ** *p* < 0.01, *** *p* < 0.001.

To unravel the potential mechanism that led to different immunotherapeutic outcomes and prognostic signatures, we further probed into the TME landscape of the risk model. As Figure 6A shows, the low-risk group possessed increased infiltration of activated myeloid dendritic cells, B cells, and CD8+ T cells. ssGSEA also found that the low-risk group had significantly higher levels of immune function, including the immune checkpoint, T cell stimulation, and immune promotion scores (Figure 6B and Figure S4A). Through the results of a Hallmark enrichment analysis, cancer-related pathways, such as P53, transforming growth factor beta (TGF-β), epithelial–mesenchymal transition, etc., were enriched in high-risk groups (Figure S4B). The results suggested that the low-risk group possessed an immune-activated TME landscape, which might be helpful for a favorable prognosis and better immunotherapeutic outcomes.

We finally explored cell–cell communication within the TME using the single-cell dataset. TME cells exhibited high clustering by cell type. Among the stromal cell types, fibroblasts accounted for the largest proportion, and T lymphocyte subgroups followed (Figure 3H). The cell–cell interaction via CellphoneDB suggested that cancer-associated fibroblasts (CAFs) were the main communicators within the TME (Figure 6C). We further investigated the ligand–receptor pairs of cytokines between CAFs and other TME cell types and found that the CCL22/CCR4 axis exhibited the most active signal communication (Figure 6D). In summary, these results implied that CAFs might contribute to the immune landscape of the TME.

## 3. Discussion

HNSCC can be classified into three TME types: hot (inflamed) tumors, cold (noninflamed) tumors, and immune-excluded tumors. Ample evidence indicates that the preexisting TME strongly influences prognosis [20], and patients with the hot-type TME benefit more from immunotherapy [21]. C/CR are chemotactic cytokines influencing the immune type of the TME by regulating the amount and distribution of infiltrating immune cells [17]. We performed a thorough analysis of the characteristics of 67 C/CR genes in HNSCC and classified patients into two clusters. Cluster two exhibited a “hot tumor” with a good prognosis. The immune cell types and immune functions of cluster two were enriched in the infiltration of cytotoxic lymphocytes (CTLs) and activation of cytolytic activity (CYT). CTLs are major anti-tumor effectors and have been regarded as the preferred tool to target tumors. The abundance of CTLs is related to the response to immunotherapy and prognosis [22,23]. Similarly, CYT is significantly upregulated with cytotoxic T cell activation and is associated with stronger immunogenicity and better clinical outcomes in several cancers [24,25,26]. Therefore, classification based on C/CR expression is reasonable and might benefit immunotherapy response and prognosis prediction.

Based on C/CR expression, we developed a risk model including six genes (CCL22, CCL26, CXCR5, CCR4, CCR7, and XCR1). Then, based on the risk model, patients were classified into low-risk or high-risk groups. First, patients in the low-risk group displayed a better response to immunotherapy. For example, we found that the low-risk group might be more sensitive to cisplatin and docetaxel, which are commonly used in neoadjuvant immunotherapy in HNSCC [27]. Additionally, it was predicted in our study that the response rate to anti-PD-L1 immunotherapy in the low-risk group would be twice that of the high-risk group. As for the prognostic prediction, we found that patients in the low-risk group displayed a longer OS and an increased median survival time compared with the high-risk group. Univariate and multivariate Cox analyses indicated that the risk score was a prognostic factor independent of age, gender, etc. Notably, predictive biomarkers based on the intrinsic characteristics of the tumor, such as TMB and gene mutation, were also introduced in our study [28]. TMB is characterized by the number of tumor gene variants and has been widely used for immunotherapy assessment [7,29]. Recent studies observed that the tumor suppressor gene TP53 mutation was associated with high TMB [30], and it might predict prognostic outcomes for HNSCC patients. We found that a high-risk score was associated with high TMB and high TP53 mutation rates, further demonstrating that the risk score was a reliable independent predictive indicator.

The high-risk group presented an immune-inactivated TME type and activated cancer-related pathways, including Myc, p53, TGF-β pathways, etc. Of note, the cell communication analysis of HNSCC showed that CAFs played a key role in cell–cell interaction among the TME. It has been reported that CAFs are a complex heterogeneous cell population [31,32]. For instance, immunosuppressive CAF subtypes mediated a positive feedback loop with Tregs, leading to the immunotherapy resistance of breast cancer [33]. Immunostimulatory CAF subtypes can reduce CD8+ T cell exhaustion and enhance the T cell cytolytic profile, contributing to favorable clinical responses to anti-PD-1 therapy of HNSCC [34]. The pan-cancer analysis also revealed that the amount of CAF subtypes with highly activated cancer hallmark pathways was positively correlated with the count of cancer cells [35]. In our study, within the C/CR ligand–receptor network of the TME, CAF-related CCR4 and CCL22 built up the most remarkable pairs. Therefore, due to the high heterogeneity of CAFs [36], different subtypes of CAFs contribute to remolding the TME and further influence the immunotherapy response and prognosis. We also found that CCL22 was commonly expressed on TME cells of HNSCC in the scRNA-seq analysis. CCL22 has been detected as frequently overexpressed in malignant cells of HNSCC [37,38,39]. The high expression of CCL22 contributed to the lymph node metastasis of HNSCC [37]. Additionally, CAF-derived IL-1β can enhance CCL22 expression in HNSCC, leading to Treg infiltration and fostering a protumor environment [39]. Our experiment also verified that CCL22 was elevated in the HNSCC cell line CNE2, indicating that the CCL22/CCR4 axis might contribute to cancer progression in HNSCC.

In conclusion, we first established a six-gene risk model based on C/CR, which achieved stratification of HNSCC patients. The risk model can simultaneously predict the immunotherapy response and prognosis. External validation and experiments were performed to verify the prediction performance and reliability of the model multidimensionally. The ability to stratify patients might be attributed to the effects of CAFs and the CCL22/CCR4 axis. A further clinical cohort is still needed to prospectively confirm whether the risk model is clinically feasible to assess therapeutic response and prognosis.

## 4. Materials and Methods

### 4.1. Data Acquisition and Consensus Clustering

A total of 67 C/CR were identified in RNA sequencing data (RNA-seq; fragments per kilobase million [FPKM] value), and corresponding clinical information was downloaded from TCGA (https://portal.gdc.cancer.gov/, accessed on 1 November 2022) [40]. Data from (https://xena.ucsc.edu/, accessed on 1 November 2022) was downloaded for CNV analysis. GSE41613, GSE65858, single-cell transcriptome files of GSE103322, and anti-PD-L1 immunotherapy cohort IMvigor210 were downloaded from the GEO. The unsupervised class was performed based on the expression level of 67 C/CR to identify their subtypes in the TCGA-HNSCC cohort. Consistency analysis was performed using the “ConsensusClusterPlus (v1.54.0)” R package [41] and repeated 1000 times; the maximum number of clusters was nine. The protein expression level was analyzed via the HPA database (https://www.Proteinatlas.org/, accessed on 15 November 2022) [42].

### 4.2. Gene Set Variation Analysis and Single-Sample Gene-Set Enrichment Analysis

Three gene sets, including “h.all.v7.4.symbols”, “c2.cp.kegg.v7.4.symbols”, and “c5.go.v7.4.symbols” were downloaded from the MSigDB database (http://www.gseamsigdb.org/gsea/msigdb/index.jsp, accessed on 5 November 2022) for gene-set variation analysis (GSVA) [43]. An adjusted *p* value < 0.05 was regarded as statistically significant. The gene ontology (GO) function analysis and the Kyoto Encyclopedia of Genes and Genomes (KEGG) pathway enrichment analysis were performed using the “GSVA” R package. The immune score, stromal score, and tumor purity were calculated by the “ESTIMATE” algorithm to estimate TME features [44]. The ssGSEA algorithm was used to quantify the relative magnitude of TME immune infiltration [45].

### 4.3. Establishment of a Risk Model

C/CR DEGs between carcinoma and adjacent tissues were identified by the “limma” R package in the TCGA-HNSCC cohort (log2|fold change (FC)| ≥ 1.0, and *p* < 0.05) [40]. A univariate Cox analysis was utilized to screen out the C/CR genes with prognostic value. A LASSO Cox regression analysis was used to develop a risk model [46].

The risk score was calculated as follows: risk score = ∑(*C* ∗ *Exp*), where C and Exp represent the risk coefficient and expression of each gene, respectively. The risk score of each patient was calculated according to the risk formula. Patients were then assigned into high- and low-risk groups according to the median risk score. A PCA was performed using the “Scatterplot3D” R package to investigate the distribution of different groups.

### 4.4. Immunotherapy Response Prediction

With the help of the pRRophetic package, we calculated the half-maximal IC50 for chemotherapeutic drugs in the TCGA-HNSCC project [47]. The difference in IC50 between the high- and low-risk groups was compared using Wilcoxon signed-rank tests. The IMvigor210 cohort, a cohort of urothelial carcinoma patients treated with the anti-PD-L1 antibody atezolizumab [48], was widely used to predict the response to immunotherapy in HNSCC [49], lung adenocarcinoma [50], ovarian cancer [51], etc. TIDE and IPS were performed to verify the response to immunotherapy [52,53].

### 4.5. Prognosis Prediction of the Risk Model

The Kaplan–Meier analysis and log-rank test were used to assess the survival outcomes between the two groups. The “survival”, “survminer”, and “timeROC” R packages were used to form the time-dependent ROC curves and calculate the AUC. Multivariate and univariate Cox regression was used to verify the independent prognostic value of risk.

### 4.6. External Cohort Validation

Two external GEO datasets (GSE41613 and GSE65858) were used as validation groups to validate the prognostic performance of the risk score. Single-cell RNA-seq datasets were analyzed using the Seurat R package (v4.0.5) [54]. All cells with fewer than 200 detected genes and genes detected in fewer than three cells were eliminated. Cell clustering and t-SNE were performed with the Seurat R package. Using Seurat’s FindAllMarkers function, we obtained markers for each cluster.

### 4.7. Immune Landscape between the Two Groups

XCELL (https://xcell.ucsf.edu/, accessed on 15 November 2022), QUANTISEQ [55], TIMER [56], MCPCOUNTER [57], EPIC (https://gfellerlab.shinyapps.io/EPIC_1-1, accessed on 15 November 2022), CIBERSORT_ABS (https://cibersort.stanford.edu/, accessed on 15 November 2022), and CIBERSORT (https://cibersort.stanford.edu/, accessed on 15 November 2022) algorithms were performed to estimate the immune cell infiltration in HNSCC. The Pearson correlation test was used to compare the correlations between the risk score and immune cell infiltration. To enable a systematic analysis of cell–cell communication, the CellPhoneDB package (www.cellphonedb.org, accessed on 20 November 2022) was performed to explore the C/CR pairs among TME cells.

### 4.8. Cell Culture and Real-Time Quantitative Polymerase Chain Reaction

Four human HNSCC cell lines (Fadu, CNE1, CNE2, and Cal-27) and one human normal oral epithelial cell line (NOK) were purchased from ATCC or obtained from the State Key Laboratory of Oral Diseases at Sichuan University. The HNSCC cell lines were cultured in Dulbecco’s Modified Eagle’s Medium (Gibco, Grand Island, NY, USA) with 10% fetal bovine serum (Gibco, Grand Island, NY, USA). The NOK cell line was cultured in keratinocyte serum-free medium (Gibco, Grand Island, NY, USA) supplemented with epidermal growth factor. RT-qPCR was performed by the Direct-zol RNA Miniprep Kit (Zymo Research, Irvine, CA, USA), SweScript RT I First Strand cDNA Synthesis Kit (Servicebio, Wuhan, China), and SYBR Green qPCR Master Mix (Servicebio, Wuhan, China). The data were computed through the 2^−ΔΔCt^ method. CCL22 primer sequences were as followed: forward primer 5′-TTACGTCCGTTACCGTCTGC and reverse primer 5′-CCACGGTCATCAGAGTAGGC. GAPDH primer sequences were as followed: forward primer 5′-GCACCGTCAAGGCTGAGAAC and reverse primer 5′-TGGTGAAGACGCCAGTGGA.

### 4.9. Statistical Analysis

R software was used for statistical analyses (version 4.0.3, http://www.R-project.org, accessed on 1 November 2022). *p* < 0.05 was considered statistically significant, and *p* values were labeled as follows: ns (*p* > 0.05), * (*p* < 0.05), ** (*p* < 0.01), and *** (*p* < 0.001).

## Data Availability

Publicly available datasets were analyzed in this study. This data can be found here: TCGA (https://portal.gdc.cancer.gov/, accessed on 1 November 2022) and GEO (https://www.ncbi.nlm.nih.gov/geo/, accessed on 1 November 2022).

## Supplementary Materials

The supporting information can be downloaded at: https://www.mdpi.com/article/10.3390/ijms24043317/s1.
