# Supplementary material for: Identification and Biological Validation of a Chemokine/Chemokine Receptor-Based Risk Model for Predicting Immunotherapeutic Response and Prognosis in Head and Neck Squamous Cell Carcinoma
Source: Int J Mol Sci. 2023 Feb 7;24(4):3317. doi: 10.3390/ijms24043317 (PMC9963044; doi:10.3390/ijms24043317)

Figure S1A. Location of CNV alteration on 23 chromosomes.

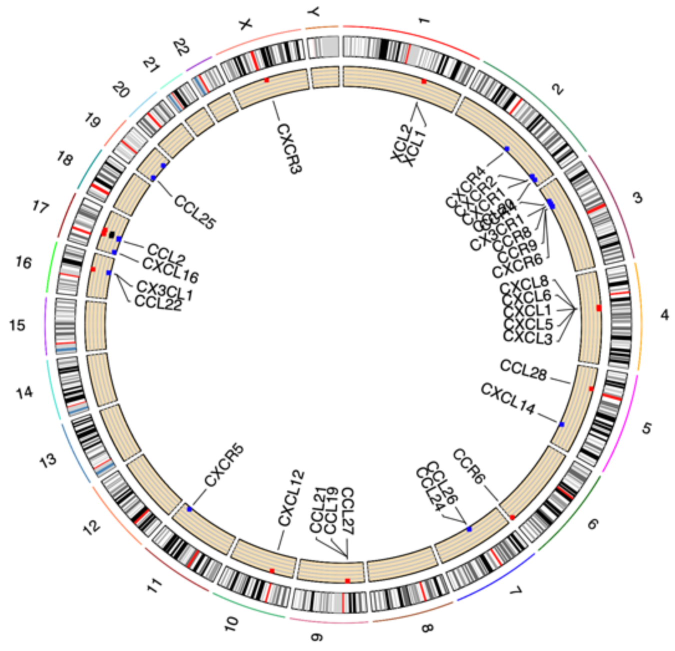

Figure S1B. Mutation frequencies of the C/CR genes.

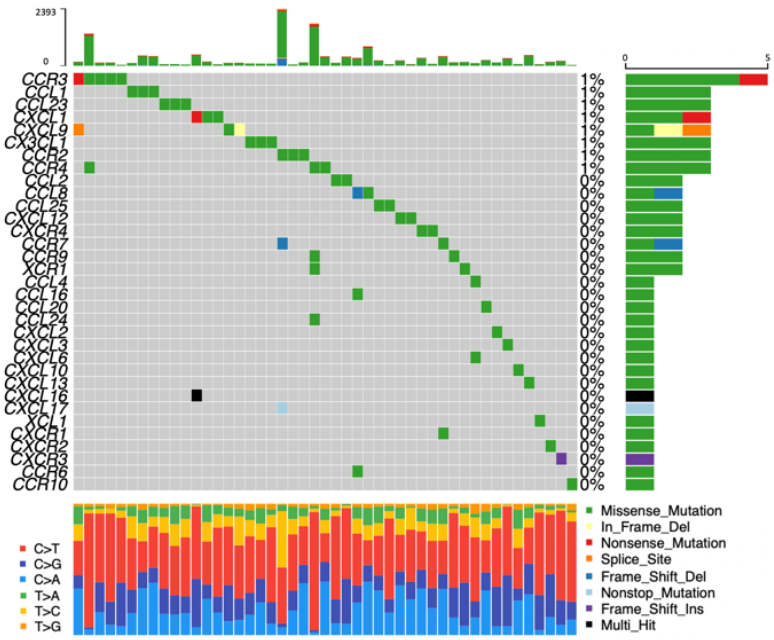

Figure S1C. Consensus clustering matrix for k = 2.

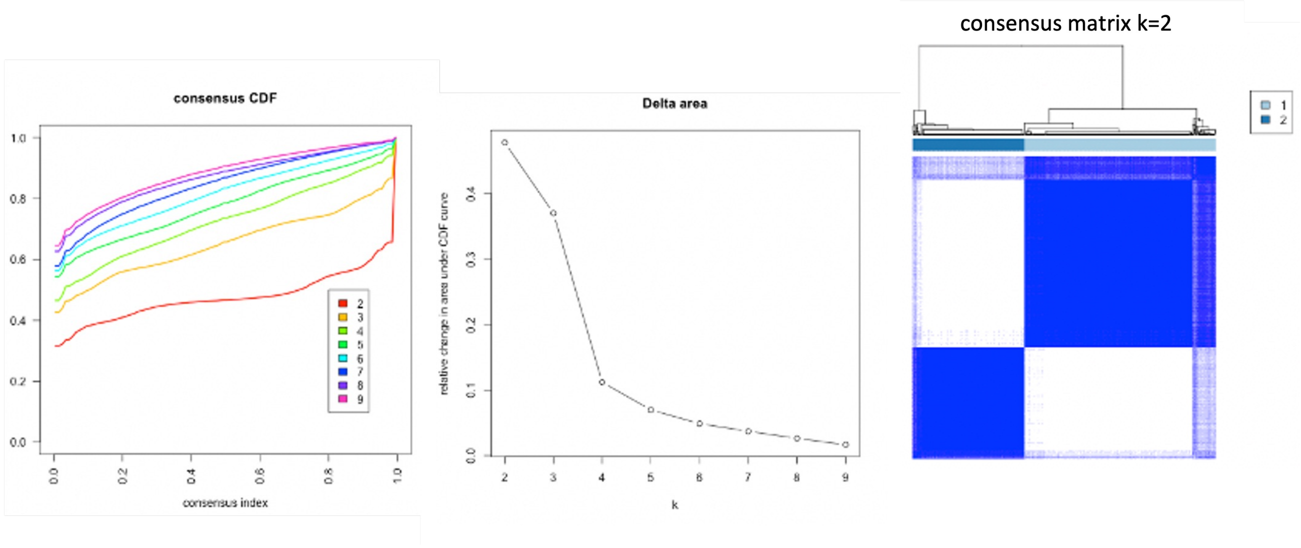

Figure S1D. Different clinicopathological features between the two clusters.

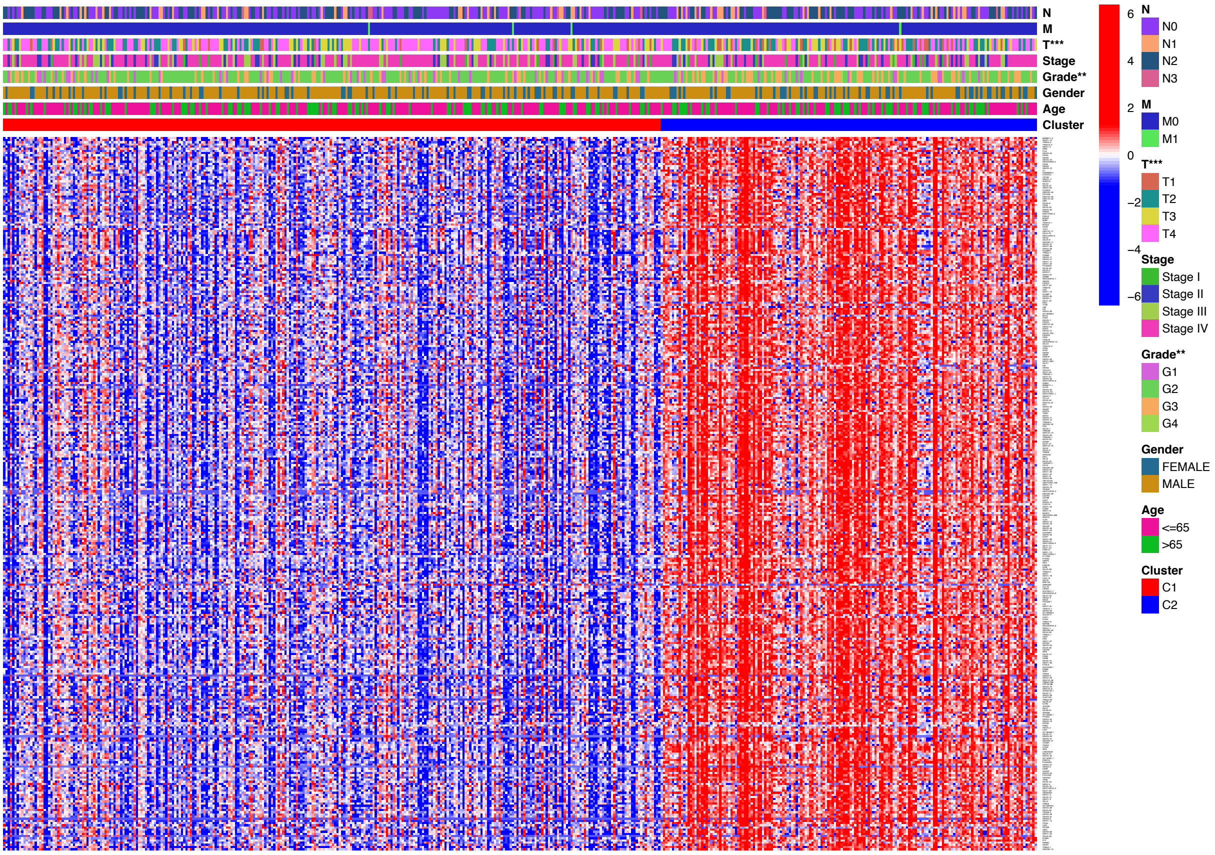

Figure S1E. Top 20 GO terms and KEGG pathways.

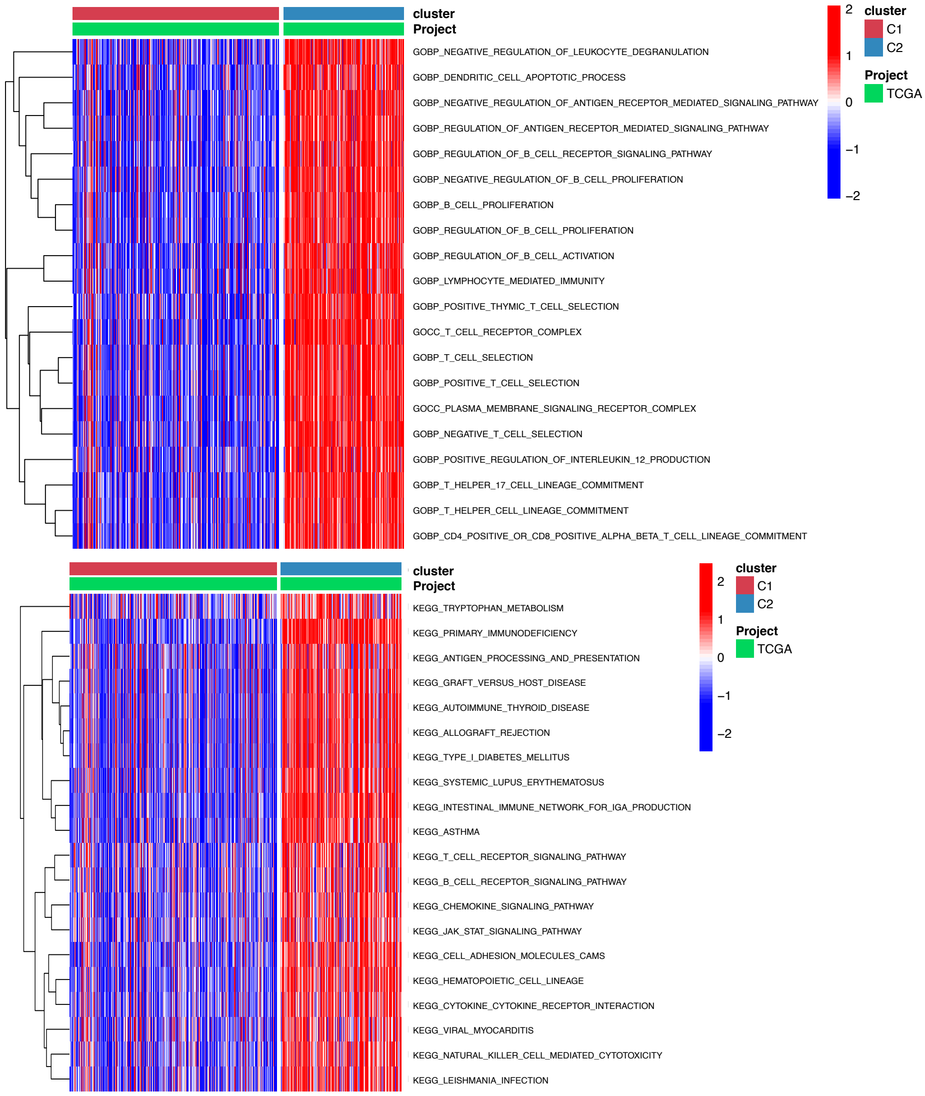

Figure S1F. Immune cell score and immune function score.

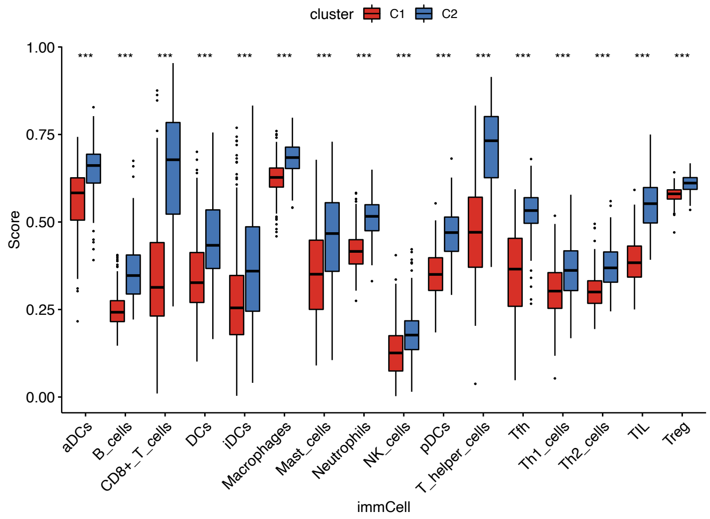

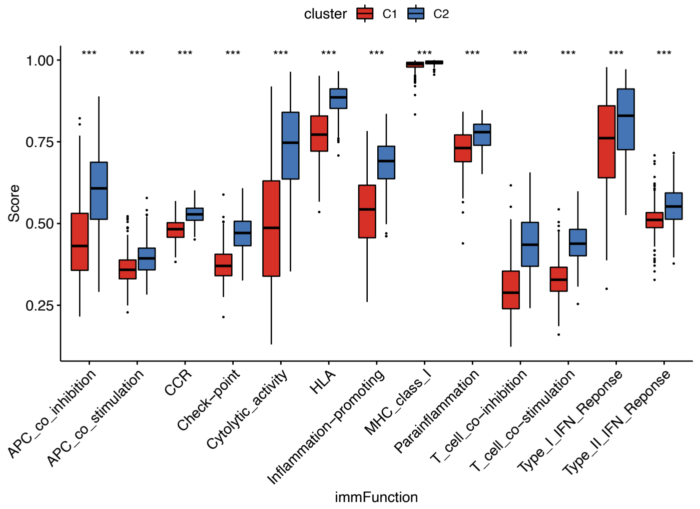

Figure S2A. LASSO coefficients profiles and LASSO regression with 10-fold cross-validation.

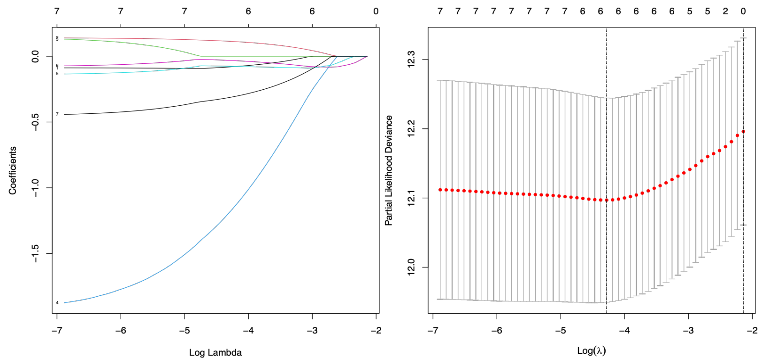

Figure S2B. Quality control of the scRNA-seq.

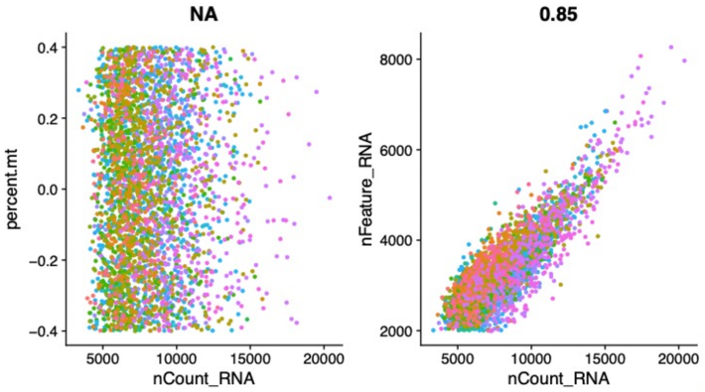

Figure S2C. Expression level of the 6 genes among 12 cell types in scRNA-seq.

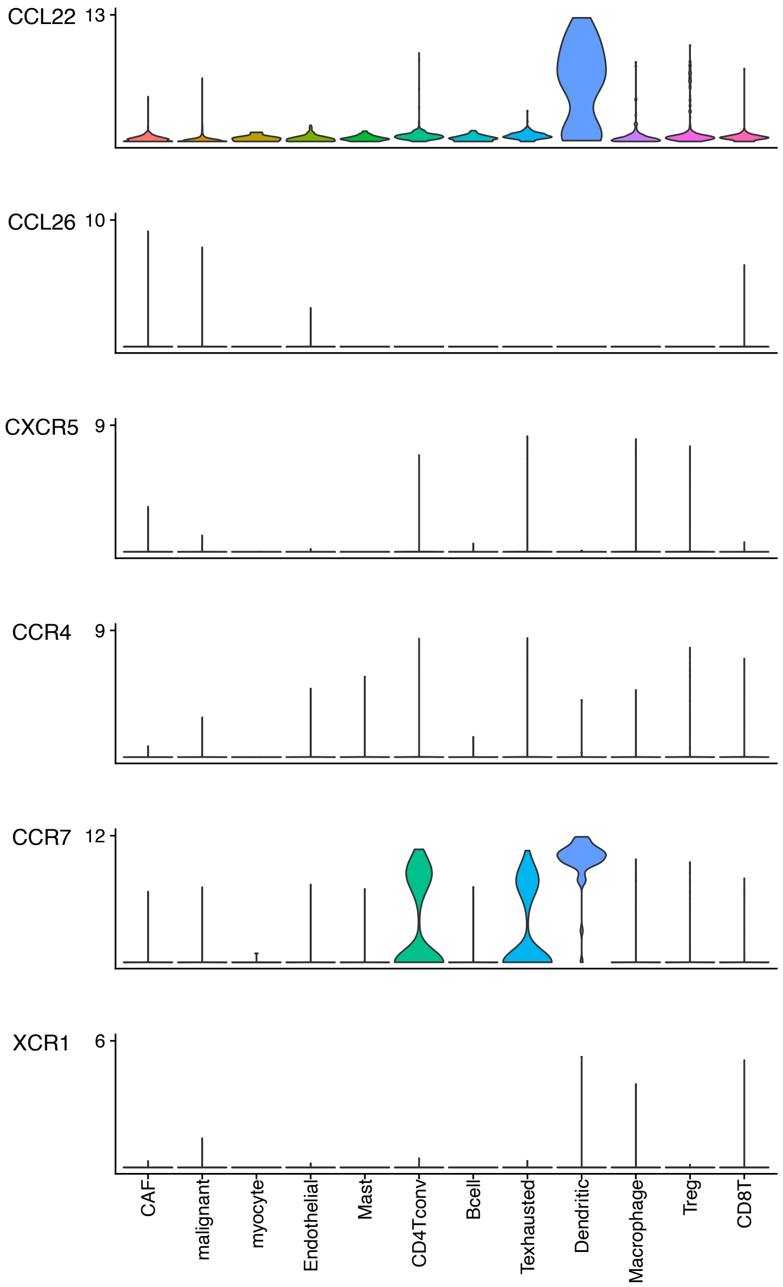

Figure S3A. The waterfall plot of tumor somatic mutation of two groups.

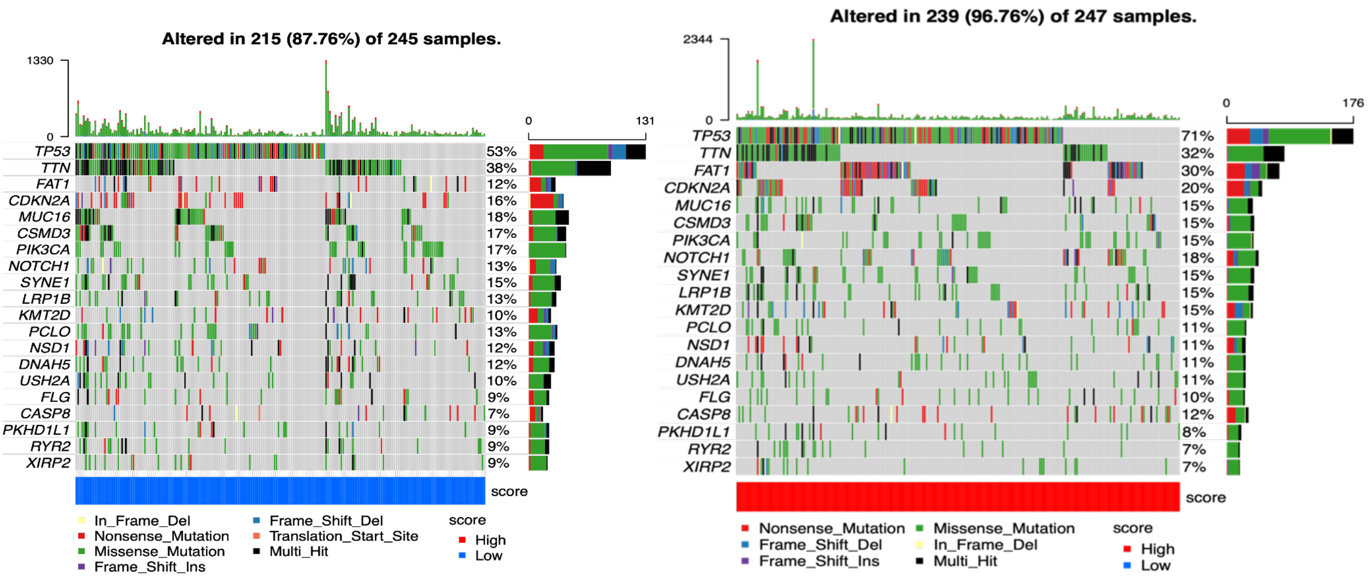

Figure S3B. Analysis of OS based on risk scores stratified by age, gender, and TNM stage.

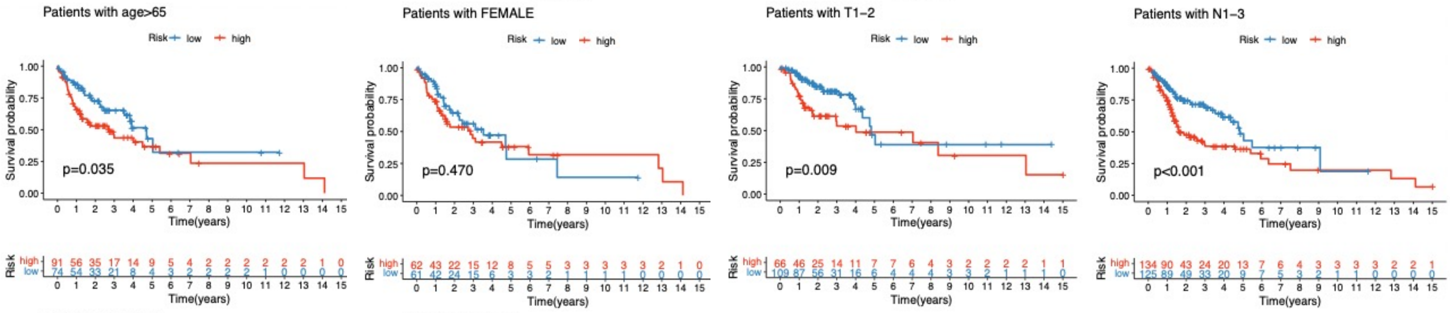

Figure S4A. KEGG pathway and immune cell score.

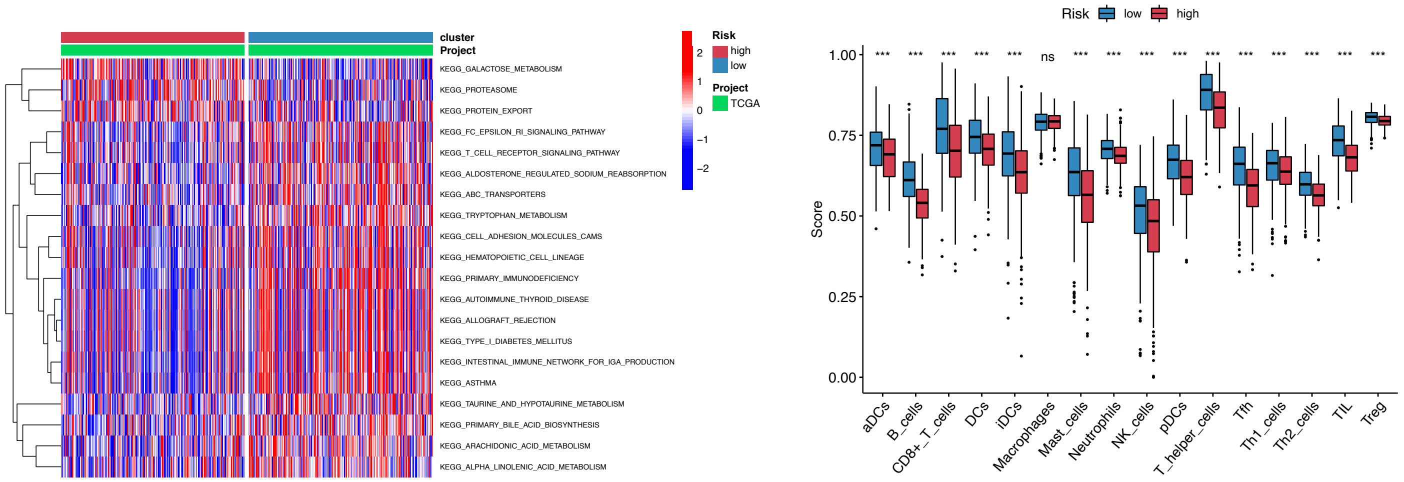

Figure S4B. HALLMARK pathway analysis.

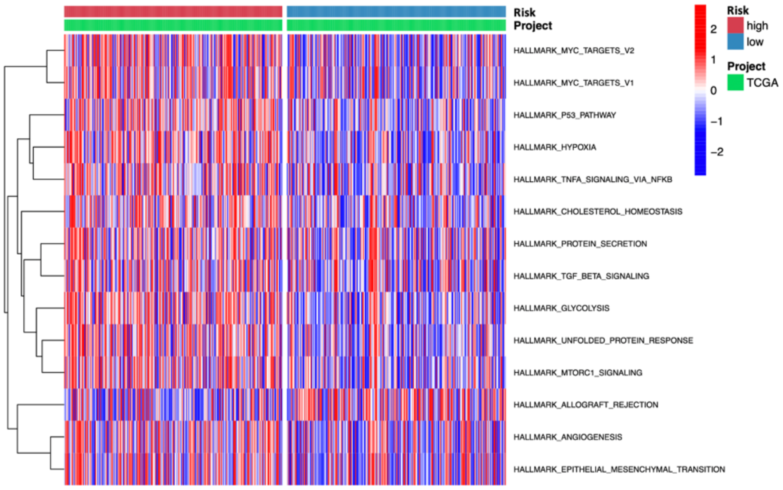

Supplement: Supplementary file 1 [file ijms-24-03317-s001.zip › ijms-2168936-supplementary.pdf]
